# Prenatal micronutrient supplementation and postpartum depressive symptoms in a pregnancy cohort

**DOI:** 10.1186/1471-2393-13-2

**Published:** 2013-01-16

**Authors:** Brenda MY Leung, Bonnie J Kaplan, Catherine J Field, Suzanne Tough, Misha Eliasziw, Mariel Fajer Gomez, Linda J McCargar, Lisa Gagnon

**Affiliations:** 1 Department of Community Health Sciences, University of Calgary, Calgary, AB, Canada; 2 Department of Paediatrics, University of Calgary, 2888 Shaganappi Trail SW, Calgary, AB, Canada; 3 Department of Agricultural, Food and Nutritional Science, University of Alberta, Edmonton, AB, Canada; 4 Department of Public Health and Community Medicine, Tufts University, Boston, MA, USA; 5 Department of Psychiatry, University of Calgary, Calgary, AB, Canada

**Keywords:** Postpartum depression, Dietary supplements, Selenium, Omega-3

## Abstract

**Background:**

Postpartum depression is a serious problem for women and their offspring. Micronutrient supplements are recommended for pregnant women because of their documented protective effects for the offspring, but their potential beneficial effects on maternal mental health are unknown. This study investigated the association between prenatal micronutrient supplementation and the risk for symptoms of postpartum depression in a longitudinal pregnancy cohort from the Alberta Pregnancy Outcomes and Nutrition (APrON) study.

**Methods:**

Participants came from a cohort of the first 600 APrON women. Supplemental nutrient intake and symptoms of depression (measured with the Edinburgh Postnatal Depression Scale (EPDS)) were collected at each trimester and 12 weeks postpartum.

**Results:**

Of the 475 participants who completed the EPDS at least twice in pregnancy and at 12 weeks postpartum, 416 (88%) scored <10 and 59 (12%) scored ≥10, where an EPDS ≥10 is considered to be “at least probable minor depression”. Mean nutrient intakes from supplements were higher in women with lower EPDS scores, particularly selenium (p = 0.0015) and omega-3s (p = 0.01). Bivariate analyses showed that several demographic and social/lifestyle variables were associated with EPDS ≥10: not having been born in Canada (p = 0.01), greater number of chronic conditions (p = 0.05), greater number of stressful life events during this pregnancy (p = 0.02), and lower prenatal and postnatal support (p = 0.0043 and p = 0.0001, respectively). Adjusting for covariates and nutrients known to be associated with postpartum depression, logistic regression showed that having a prenatal EPDS ≥ 10 increased the odds of postpartum depressive symptoms (second and third trimester OR = 3.29, 95% CI = 1.55 - 7.01, p = 0.004 and OR = 4.26, 95% CI = 2.05 - 8.85, p < 0.0001, respectively), while prenatal supplemental selenium (per 10 mcg, OR = 0.76, 95% CI = 0.74 - 0.78, p = 0.0019) and postnatal social support (OR = 0.87, 95% CI = 0.78 - 0.97, p = 0.0015) were protective.

**Conclusions:**

Multiple factors, including supplementary selenium intake, are associated with the risk of postpartum depressive symptoms. Future research on dietary supplementation in pregnancy with special attention to selenium intake is warranted.

## Background

Postpartum depression is a serious mental health problem that has been estimated to affect 10 - 15% of women after the birth of a child [1], and can occur anywhere from shortly after birth to one year postpartum. A review by Gavin and colleagues stated the prevalence may be as high as 19% within the first three months of giving birth [2]. The difference in rates may be due to the diagnostic criteria, timing of screening, and screening instruments used [1]. Regardless of the precise prevalence rate, it is clear that postpartum depression poses a significant public health issue because of its impact not only on the lives of the women themselves, but also on their children’s growth and development (cognitive, social, behavioural) [3,4].

A number of social and biological factors are known to be associated with an increased risk for developing postpartum depression. Social risk factors include a history of depression/anxiety, lack of a marital partner, marital difficulties, poverty, and lack of social support [5], as well as family violence, life stress, and substance abuse [6]. Biological risk factors associated with postpartum depression include hormonal influences [7] and nutrient deficiencies from malnutrition or poor diet quality [8-10]. Research has shown a number of nutrient deficiencies associated with depression in the non-pregnant or postpartum population, including folate and vitamin B12 [11,12] , calcium [12,13], iron [11,12,14], selenium [11,15], zinc [11,16] and omega-3 polyunsaturated fatty acids [11,17-23]. Postpartum depression is not qualitatively different from depression that is not linked to pregnancy, but only recently has research on the role of nutrients in postpartum depression begun to emerge [24]. A summary has been published elsewhere describing some of the known biochemical effects of these nutrients on brain and neurological function, pointing toward the neurophysiological mechanisms by which insufficient nutrients could influence mood [24].

It is well-documented that proper nutrition during pregnancy is vital to the health of a woman and her fetus [25,26]. Today, it is common for health professionals to recommend that pregnant women take micronutrient supplements, the benefits of which have been established for the offspring. For instance, a meta-analysis by Goh and colleagues reported that offspring of women who took prenatal multivitamins were less likely to have leukemia (odds ratio (OR)=0.61, 95% confidence interval (CI)=0.50-0.74), pediatric brain tumors (OR=0.73, 95% CI=0.60-0.88) and neuroblastoma (OR=0.53, 95% CI=0.42-0.68), compared to children whose mothers did not take supplements [27]. Another meta-analysis found prenatal supplements to be associated with decreased risk for a number of congenital anomalies (e.g. cardiovascular defects, limb defects, urinary tract anomalies, and cleft palate or oral cleft anomalies), in addition to neural tube defects [28]. A meta-analysis of research in developing countries showed that multiple micronutrient supplementation was more effective than iron and folic acid supplementation at reducing the risk of low birth weight (RR=0.86, 95% CI=0.79-0.93) and of small size for gestational age (RR=0.85; 95% CI=0.78-0.93), but had no effect on perinatal mortality [29].

These results demonstrate important protective effects for the offspring from maternal use of prenatal multiple micronutrient supplementation. The effect of prenatal supplementation on maternal mental health, however, has not been established. The *purpose* of this study was to investigate the association between prenatal micronutrient supplementation and the risk for postpartum depressive symptoms in pregnant women from the Alberta Pregnancy Outcomes and Nutrition (APrON) study. We evaluated the nutrients ingested through supplements to determine whether any individual supplementary nutrients were associated with postpartum depressive symptoms as measured by the Edinburgh Postnatal Depression Scale (EPDS).

## Methods

### Study design and participants

**Figure 1:**
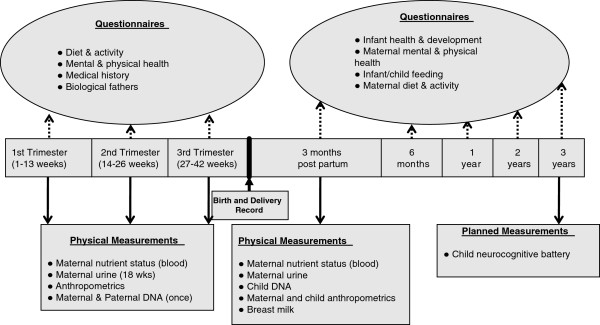
APrON timeline.

Participants for this study are the first 600 pregnant women from the APrON study, which is a longitudinal prospective study in Alberta, Canada. Participants were at least 16 years old with gestational age ≤27 weeks. Women must be in the first (T1) or second (T2) trimester to be in this study; we did not include any woman who was 28 weeks or beyond. Non-English speakers, known drug and alcohol abusers, and those planning to move out of the region within 6 months were excluded. Data were collected using questionnaires and interviews at each trimester and 12 weeks postpartum. Windows for data collection were defined *a priori* as week 10 ±2 for first trimester, week 18 ± 2 for second trimester, and week 32 ± 2 for third trimester. Every attempt was made to meet these specific timepoints as closely as possible (see Figure 1). Details of recruitment and data collection are available elsewhere [30]; also refer to the APrON website at http://www.ApronStudy.ca.

#### Participant background and covariates

Data on sociodemographic background and variables such as stressful life events and perceived social support known to be associated with increased risk for postpartum depression were collected using a questionnaire developed specifically for the APrON study (see Table 1). Perceived social support was measured with questions from the National Population Health Survey (NPHS) – Social Support section (Statistics Canada, 1994/95 & 1996/97 cycles) [31] with response options modified for the APrON study. The NPHS-Social Support section is comprised of four statements about having “someone to confide in”, “someone one can count on in a crisis”, “someone one can count on for advice”, and “someone who makes one feel loved and cared for”, with Yes/No response options. Guided by expert advice from team members, the APrON study kept the wording of the statements, but modified the response options to five possible answers: none of the time, a little of the time, about half of the time, most of the time, all of the time. Each response option was then assigned a numerical value, “none of the time” was assigned a value of 0 and ranged up to a value of 4 for “all of the time”. By summing the scores of each statement, an overall score of 16 indicated the highest possible level of perceived social support, while an overall score of 0 was the lowest possible level of perceived social support.

**Table 1:** Associations between socio-demographic characteristics of women and their postpartum EPDS

**Characteristic**	EPDS<10*	EPDS≥10*	p-value**	Odds ratio*** (95% CI)
***Demographics***				
Age (mean (95% CI))	31.2 (30.8 - 31.6)	31.6 (30.3 - 32.8)	0.51	1.0 (0.9 - 1.1)
BMI (mean (95% CI))	24.1 (23.6 - 24.5)	24.2 (22.9 - 25.4)	0.90	1.0 (0.9 - 1.0)
Marital status (n (%))				
Married/common-law	406 (88.0)	55 (11.9)	0.17	1.0
Single/divorced	9 (75.0)	3 (25.0)		2.4 (0.6 - 9.3)
Education (n (%))				
High school	36 (87.8)	5 (12.2)	0.96	1.0
Trade/undergrad	286 (87.7)	40 (12.2)		0.6 (0.1 - 7.0)
Graduate	93 (87.7)	13 (12.2)		0.6 (0.1 - 5.9)
Household Income (n (%))				
< $20K	5 (83.3)	1 (16.7)	0.13	1.0
$20-$39K	12 (80.0)	3 (20.0)		1.2 (0.1 - 15.1)
$40-$69K	47 (78.3)	13 (21.7)		1.4 (0.1 - 12.9)
$70-$99K	107 (87.7)	15 (12.3)		0.7 (0.1 - 6.4)
≥$100K	236 (90.1)	26 (9.9)		0.5 (0.1 - 4.9)
Born in Canada (n (%))				
Yes	349 (89.9)	39 (10.0)	0.01	1.0
No	63 (79.7)	16 (20.2)		2.3 (1.2 - 4.3)
Ethnicity (n (%))				
Caucasian	363 (88.3)	48 (11.6)	0.30	1.0
Non-causasian	51 (83.6)	10 (16.4)		1.5 (0.7 - 3.1)
# of children (n (%))				
0	57 (87.7)	8 (13.3)	0.28	1.0
1-2	172 (88.2)	23 (11.8)		1.0 (0.5 - 1.9)
3-4	4 (66.7)	2 (34.3)		7.3 (1.0 - 53.7)
***Social/lifestyle***				
# of chronic conditions (n (%))				
0	206 (85.1)	36 (14.9)	0.05	1.0
1	91 (91.9)	8 (8.1)		0.5 (0.2 - 1.1)
2	15 (78.9)	4 (21.0)		1.5 (0.5 - 4.8)
3	5 (62.5)	3 (37.5)		3.4 (0.8 – 15.0)
# of life event stresses (n (%))				
During this pregnancy				
0	207 (89.6)	24 (10.4)	0.02	1.0
1-2	184 (88.0)	25 (11.9)		1.2 (0.6 - 2.1)
>3	24 (72.7)	9 (27.2)		3.2 (1.3 - 7.7)
12 mos before pregnancy				
0	196 (89.9)	22 (10.1)	0.08	1.0
1-2	149 (88.2)	20 (11.8)		1.2 (0.6 - 2.3)
>3	13 (72.2)	5 (27.8)		3.4 (1.1 - 10.5)
Before age 17				
0	126 (89.3)	15 (10.6)	0.06	1.0
1-2	224 (89.2)	27 (10.7)		1.2 (0.6 - 2.6)
>3	11 (68.7)	5 (31.2)		3.8 (1.1 - 12.5)
Social support (mean (95% CI))				
Prenatal	14.8 (14.7 - 15.0)	13.8 (13.1 - 14.5)	0.0043	0.8 (0.7 - 0.9)
Postpartum	14.7 (14.5 - 14.9)	13.0 (12.2 - 13.8)	0.0001	0.8 (0.7 - 0.9)
***EPDS score by time points***				
(mean (95% CI)				
Time A (1^st^ trimester)	4.8 (4.1 - 5.6)	8.0 (6.5 - 9.5)	0.0006	1.31 (1.11-1.55)
Time B (2^nd^ trimester)	5.0 (4.7 - 5.4)	8.4 (7.2 - 9.5)	<0.0001	1.21 (1.13-1.30)
Time C (3^rd^ trimester)	4.5 (4.2 - 4.8)	9.0 (7.9 - 10.1)	<0.0001	1.33 (1.23-1.44)
Time E (12 weeks postpartum)	3.6 (3.4 - 3.8)	12.1 (11.4 - 12.9)	<0.0001	n/a

* EPDS≥10 = “at least probable minor depression”. The frequency calculations differed by variable because of missing values in some cases (e.g. participant did not answer a question).

**Assessment of associations with Chi-square test for categorical variables or t-test for continuous variable.

***OR from univariate logistic regression.

#### Prenatal nutrient intake from supplements

The Supplement Intake Questionnaire was developed for APrON to assess natural health product (NHP) intake. The structure of the Supplement Intake Questionnaire was adapted from questionnaires used in other studies of supplement intake, such as the National Cancer Institute and the Canadian Community Health Survey [32,33]. The Supplement Intake Questionnaire was pilot tested with a panel of nutrition experts as well as 50 pregnant women to determine whether it captured NHP data efficiently and accurately. Wording, formatting, and other modifications were made based on the feedback from the pilot study.

The final version of the Supplement Intake Questionnaire consisted of three sections: 1) section 1 listed options for multivitamins/minerals, 2) section 2 listed single nutrient supplements, and 3) section 3 consisted of herbal products, probiotics, homeopathic remedies, and other products such as amino acids, essential fatty acids and traditional medicines. Commonly used formulations were presented in a checklist. For products used by participants but not in the checklist, space was provided to record the name and manufacturer of the NHP as well as the Natural Product Number (NPN) or Drug Identification Number (DIN). Where possible, the products were verified using their NPNs and DINs against those within Health Canada’s Natural Health Products Database, to confirm their formulations; then they were entered into the APrON database. A NPN or a DIN is assigned to all NHPs that have met standards for quality, safety and efficacy set by Health Canada [34]. Products that did not have a NPN or DIN were verified by obtaining ingredients from product labels or by finding the information from the manufacturer/supplier websites.

At each visit during pregnancy and postpartum, women were asked to describe in detail the quantity (i.e., frequency of intake and dosage) and type (e.g., prenatal multivitamins) of NHP consumed, using open-ended questions. To determine dose, women were asked “how much do you take?” and for frequency, women were asked “how often do you take it?” Typical responses were daily or times per week (e.g., 5 x/week). There was no menu of responses from which to choose. For example, if a woman said she consumed 700 mg/week of a nutrient, it was calculated as 100mg/day for that specific trimester. The daily value was calculated for the specific trimester, and only for that trimester, and not extrapolated for the entire pregnancy.

A trained Research Assistant conducted the interview with each woman to elicit the information. All information provided by the participants was reviewed with them to ensure the items were correctly recorded. Women were also asked to bring in bottles and other containers of the supplements they were taking. Brand names, individual nutrients and their amounts, as well as dosage (i.e. number of pills/capsules/tablets taken per day) were recorded. Prior to each visit, an email was sent to the women, reminding them to bring in the NHP container(s) and other materials to the next clinic visit. When a woman did not bring in the container(s) of the NHPs, the research assistant followed the visit with a phone call to obtain the information.

For the purpose of this study, only supplements of vitamins, minerals, and fatty acids were analyzed. Supplement data was collected a minimum of two times, and maximum of three times during pregnancy. To obtain an average intake of each nutrient in the supplements, reported intake was averaged over the number of times data were collected. For example, if information on vitamin D supplement intake was collected at each trimester (i.e., three times), then the total amount consumed was divided by three; if information was collected at only two times, then the total was divided by two. The nutrients chosen for analysis in this study (listed in Table 2) were informed by the literature that has previously demonstrated a relationship with mood [8]. The Recommended Dietary Allowance (RDA) values from the Institute of Medicine [35] were used as the reference values for calculating the percentage of women above or below RDA for consuming the individual micronutrients from supplements. The comparison to RDA in this study did not include nutrients from dietary intake, but from supplement intake only.

**Table 2:** Mean (SD), range and percentage below and above RDA for prenatal micronutrient intake from supplementation in pregnant women with postpartum EPDS<10 & EPDS≥10

**Nutrient**	**EPDS<10**	**EPDS≥10**	p-value*
	**(n=416)**	**(n=59)**	
Vitamin B1 (mg)			
Mean (SD)	5.5 (14.7)	3.5 (8.1)	0.12
Range	0 – 103	0 – 62.5	
Below RDA (n (%))	55 (84.6)	10 (15.4)	
Above RDA (n (%))	361 (88.1)	49 (11.9)	
Vitamin B3 (mg)			
Mean (SD)	22 (23)	19 (10)	0.10
Range	0 – 335	0 – 66	
Below RDA (n (%))	55 (82.1)	12 (17.9)	
Above RDA (n (%))	361 (88.5)	47 (11.5)	
Vitamin B6 (mg)			
Mean (SD)	9.1 (15.9)	7.8 (10.7)	0.43
Range	0 – 110	0 – 62.5	
Below RDA (n (%))	23 (79.3)	6 (20.7)	
Above RDA (n (%))	363 (88.1)	49 (11.9)	
Vitamin B9 (mcg)			
Mean (SD)	1259 (924)	1297 (962)	0.78
Range	0 – 6000	0 – 5000	
Below RDA (n (%))	30 (81.1)	7 (18.9)	
Above RDA (n (%))	386 (88.1)	52 (11.9)	
Vitamin B12 (mcg)			
Mean (SD)	31 (116)	18 (65)	0.23
Range	0 – 1210	0 – 502	
Below RDA (n (%))	19 (79.2)	5 (20.8)	
Above RDA (n (%))	397 (88.0)	54 (12.0)	
Vitamin D (IU)			
Mean (SD)	618 (539)	567 (512)	0.48
Range	0 – 4200	0 – 2750	
Below RDA (n (%))	292 (87.7)	41 (12.3)	
Above RDA (n (%))	124 (87.3)	18 (12.7)	
Iodine (mcg)			
Mean (SD)	173 (68)	158 (76)	0.15
Range	0 – 636	0 – 330	
Below RDA (n (%))	227 (85.7)	38 (14.3)	
Above RDA (n (%))	189 (90.0)	21 (10.0)	
Iron (mg)			
Mean (SD)	33 (20)	32 (20)	0.84
Range	0 – 127	0 – 87	
Below RDA (n (%))	68 (82.9)	14 (17.1)	
Above RDA (n (%))	348 (88.5)	45 (11.5)	
Magnesium (mg)			
Mean (SD)	67 (65)	59 (39)	0.19
Range	0 – 1025	0 – 208	
Below RDA (n (%))	415 (87.5)	59 (12.5)	
Above RDA (n (%))	1 (100.0)	0	
Selenium (mcg)			
Mean (SD)	25 (17)	19 (13)	0.0015
Range	0 – 125	0 – 45	
Below RDA (n (%))	401 (87.2)	59 (12.8)	
Above RDA (n (%))	15 (100.0)	0	
Zinc (mg)			
Mean (SD)	13 (8)	12 (8)	0.38
Range	0 – 48	0 – 32	
Below RDA (n (%))	233 (87.9)	32 (12.1)	
Above RDA (n (%))	183 (87.1)	27 (12.9)	
Omega-3 (mg)			
Mean (SD)	180 (440)	90 (208)	0.01
Range	0 – 4050	0 – 1000	
Below RDA (n (%))	RDA not determined	RDA not determined	
Above RDA (n (%))			

Note: RDA (Recommended Dietary Allowance) for pregnant women is “the average daily nutrient intake level sufficient to meet the nutrient requirement of nearly all (97– 98%) healthy individuals in a particular life stage and gender group” as defined by the Institute of Medicine (55). The percentages below and above RDA are calculated from supplements alone and does not account for dietary intake.

SD = Standard deviation.

* p values derived from t-tests.

#### Depression symptom measure – EPDS

The EPDS is a 10-item scale that measures mood, and requires about five minutes to complete. It has been widely validated, widely utilized, has a moderate to good reliability and test-retest reliability and has a good to moderate correlation with other depression measures [36]. It has a maximum score of 30; a score of 10 or more indicates possible depression of varying severity. The standardized and validated EPDS has been used extensively worldwide, and has been evaluated for its psychometric rigor [37-39]. It has been found to have high sensitivity and specificity (79% and 97% for first trimester at cut-off of 11, 70% and 96% for second trimester with cut-off of 10, and 76% and 94% for third trimester with cut-off of 10 [40]) in identifying those at risk of, or potentially suffering from prenatal depression.

The EPDS has been used for both clinical and research purposes, and cut-off scores have been obtained through empirical assessment [40,41]. These cut-offs provide rates of probable depression for referral or treatment, and can also be used to track the course of depression from antenatal to postpartum periods. As with other screening measures, sensitivity and positive predictive value may be variable when used in selected populations versus community populations [36,38]. For the purpose of this study, we used a cut-off score of 10 for probable depression [41]. Using the sample size of 397 we had 90% power to detect a relative risk of 1.5 or more for EPDS >10 in women consuming lower vs. adequate RDA of a nutrient from supplements.

#### Assessing predictors of depressive symptoms on the EPDS

From the first model, only *born in Canada* was statistically significant (OR = 2.27, 95% CI = 1.20 - 4.31, p = 0.012). In the second model, only postpartum social support was statistically significant (OR = 0.83, 95% CI = 0.72 - 0.95, p = 0.007). A third model with the nutrient variables resulted in only selenium being statistically significant (OR = 0.97, 95% CI = 0.94 - 0.99, p = 0.030). Although omega-3 was not significant in the logistic regression (OR = 1.00, 95% CI = 0.99 - 1.00, p = 0.07), given the strong association of omega-3 with depressive symptoms, both selenium and omega-3 were included in the next model with the significant variables from the first two models.

The fourth model was constructed to include *born in Canada*, *prenatal and postnatal social support*, *prenatal EPDS* (at 2^nd^ and 3^rd^ trimesters) and *prenatal omega-3* and *selenium* supplement. Prenatal EPDS scores were included because the literature has reported that prenatal depressive symptoms were predictive of postpartum depressive symptoms. Born in Canada, prenatal social support, and omega-3 were removed from the fourth model as they exceeded the pre-set p value of 0.05. Potential confounding of the remaining model by sociodemographic and lifestyle variables was assessed. No meaningful differences were found between the crude and adjusted ORs. Therefore, the final model consisted of predictors that were statistically significant (see Table 3). Hosmer and Lemeshow's goodness-of-fit test resulted in chi^2^(7) = 8.08, and p = 0.33, indicating that our model fits the data well. The mean VIF = 1.13, with VIF for individual variable ranged from 1.03 to 1.17 (and tolerance from .84 to .97), indicating that multicollinearity is not likely to be a problem in the model.

**Table 3:** Full and final models of predictors for EPDS depressive symptoms (EPDS≥10)

**Predictor**	**Odds ratio**	**95% CI**	**p-value**
*Model 1*			
Age	.98	.88-1.09	.69
Parity	.98	.56-1.71	.94
Marital status	.49	.19-1.24	.14
Education	.98	.63-1.51	.92
Income	5.32	.85-33.35	.07
Born outside Canada	2.41	.81-7.19	.11
Ethnic background	.73	.19-2.79	.65
BMI	1.00	.88-1.15	.90
*Model 2*			
Life stressors during this pregnancy	1.21	.87-1.69	.26
Life stressors 12 mons before this pregnancy	1.08	.77-1.50	.65
Life stressors prior to age 17	1.22	.85-1.75	.29
Social support	.85	.74-.97	.015
*Model 3*			
Vitamin B1	.98	.94-1.02	.33
Vitamin B3	.99	.97-1.01	.41
Vitamin B6	1.01	.98-1.05	.41
Vitamin B9 (folic acid)	1.00	.99-1.00	.96
Vitamin B12	1.00	.99-1.00	.76
Vitamin D	1.00	.99-1.00	.92
Iodine	.99	.99-1.00	.11
Iron	1.00	.98-1.01	.91
Magnesium	.99	.99-1.00	.37
Selenium	.97	.95-0.99	.03
Zinc	.98	.95-1.02	.41
Omega 3	1.00	.99-1.00	.20
*Final model*			
Prenatal EPDS (2nd trimester)	3.29	1.55 – 7.01	0.004
Prenatal EPDS (3rd trimester)	4.26	2.05 – 8.85	0.000
Postpartum Social Support	0.87	0.78 - 0.97	0.015
Prenatal Selenium supplement intake (per 10 mcg)	0.76	0.74 - 0.78	0.019

#### Missing values

To assess whether the 125 “missing” postpartum EDPS participants differed from those who responded, various statistical tests were used, including 1) exclusion of cases with missing postpartum EPDS, 2) re-categorization of missing as “not depressed”, 3) re-categorization of missing as “depressed”, 4) last observation carried forward using third trimester data, 5) creation of a category called “missing” to see what predicted “missing”, and 6) comparison of participant characteristics with those without postpartum EPDS data.

#### Statistical methods

All statistical analyses were performed using STATA11 software. Values were expressed as mean and standard deviation, range, or proportions (n and %). Comparisons between groups (those having EPDS scores <10 and those with EPDS scores ≥10) were assessed for statistical significance by a Chi-square test for categorical variables and a t-test for continuous variables. Bonferroni correction was used for multiple test comparisons. Several logistic regression models were constructed to identify predictors of postpartum depression. Using multivariate logistic regression, the first model consisted of all the demographic variables; model two included social/lifestyle variables plus the significant variable from model one. The third model assessed nutrients from supplement intake (listed in Table 2), and the fourth model incorporated significant (and close to significant) variables from models one, two, and three (all the models with the covariates and nutrients and their respective outputs are provided in Table 3). For the final model, we tested the fit of the model with Hosmer and Lemeshow's goodness-of-fit test, and we computed the variance inflation factor (VIF) to test for collinearity. Odds ratios (OR) and the 95% CI for predictors of the EPDS depressive symptoms were calculated. A two-tailed p-value of 0.05 was considered statistically significant.

#### Ethics approval

The APrON study was approved by the Conjoint Health Research Ethics Board at the University of Calgary, and the Health Research Ethics Board at the University of Alberta. Each participant signed a consent form at the first clinic visit.

## Results

Of the 600 pregnant women in the study cohort, 475 completed the EPDS questionnaire at least twice during pregnancy and at 12 weeks postpartum. Of the 125 with no EPDS data postpartum, nine miscarried, 36 withdrew from the study, 38 were non-respondent (i.e., could not be contacted), and 36 did not complete the EPDS. No differences in characteristics were found between those with postpartum EDPS data and those without.

Of the 475 available participants, 416 (88%) scored <10 on the EPDS and 59 (12%) scored ≥10. For demographic characteristics, women not *born in Canada* were more likely to have an EPDS ≥10 (p = 0.01); no other potential covariates were statistically significant (see Table 1). For the social/lifestyle characteristics, the *number of chronic conditions, stressful life events during this pregnancy,* and *pre/post-natal support* were all statistically significant (see Table 1); i.e. EPDS was associated with these characteristics. Women with higher number of stressful life events 12 months before pregnancy and before the age of 17 were more likely to have EPDS ≥10, with p-values close to significance (p = 0.08 and 0.06 respectively). Not presented were regressions for prenatal depression and the key nutrients found to be significantly associated with postpartum depression: the results (not shown) were that selenium was not a predictor of prenatal depression at second or third trimesters. Although not statistically significant, a trend was shown for women being single/divorced, had lower income, or had higher number of children to be associated with EPDS ≥10.

### Nutrient data

Almost all women (99%) took some type of micronutrient supplement during the prenatal period. For this cohort, the nutrients most commonly consumed were vitamins B6, B9 (folate), B12, and E, with more than 90% above RDA. The supplement taken the least was omega-3, where 68.5% of women with EPDS <10 and 78.0% of women with EPDS ≥10 did not take any omega-3. The mean intake of *selenium* and *omega-3* differed significantly (p = 0.0015 and 0.01, respectively) between women with EPDS <10 and those with EPDS ≥10. As well, the mean intakes for all the other nutrients listed in Table 2 were more likely to be higher in women with EPDS <10 than those with EPDS ≥10, although not statistically significant. In fact, the upper ranges for intake were higher in almost all nutrients (except for folic acid) in women with EPDS <10 than women with EDPS ≥10. Finally, all the other nutrients listed (except vitamin D and zinc) showed an overall nonsignificant trend for those consuming below RDA levels to be more likely to have EPDS ≥10 (see Table 2). Supplement intake for many of the nutrients was not normally distributed. SIQ data was available for n=136 in the first trimester, n=575 in the second trimester and n=516 in the third trimester.

Table 3 showed the (statistically significant) predictors for postpartum depressive symptoms (i.e. EPDS ≥10) from the final model. Selenium use above RDA levels and postnatal social support reduced the odds of the EPDS ≥10, while prenatal EPDS (at 2^nd^ and 3^rd^ trimesters) increased the risk of EPDS ≥10.

## Discussion

The results of this research support previous studies on the nutritional and social factors associated with depression [5,42,43]. In this study, women with an EPDS score ≥10 were more likely to be born outside of Canada; to report having more chronic health conditions, more life stress and less social support during the current pregnancy; and to have consumed fewer micronutrients from supplements, most notably selenium and omega-3. The logistic regression showed that prenatal EPDS ≥10 (at second and third trimesters) increased the odds of postpartum depressive symptoms, while prenatal selenium intake from supplements and postnatal social support were protective (decreased the odds) of postpartum depressive symptoms. Of the nutrients evaluated, all intakes were likely to be higher in women with EPDS <10 than those with EPDS ≥10.

The role of selenium in relation to mood is not as well studied as some other nutrients (e.g. omega-3s, folate, and zinc) that have been associated with depression. A recent nested case–control study by Pasco and colleagues found low intake of selenium (<8.9 μg/MJ/day) was associated with almost a three-fold increase in the likelihood of major depressive disorder (OR 2.95, 95% CI 1.00-8.72) after adjusting for age and socio-economic status [44]. A randomized trial by Mokhber and colleagues reported significantly lower mean EPDS scores in the group of primigravid pregnant Iranian women, aged 16 to 35, taking selenium supplements compared to a comparison group after controlling for sociodemographic and health history variables [42]. Hawkes and Hornbostel in a study of eleven healthy men found lower selenium status associated with worse mood scores [45].

This study reinforced the importance of social support for pregnant women in the prenatal and postnatal periods, the benefits of which have been well-documented for the reduction of the risk and symptoms of mood disorders and depression [43,46,47]. While there is little research into the mechanism by which environmental factors such as social support affect brain biochemistry in humans, animal studies indicate that improving external stimuli through an enriched environment may reverse the effects of stress-related events, at the behavioral, endocrine, and biochemical levels [48].

The findings from this study also revealed the need for evaluation for prenatal depressive symptoms. While there are programs in many jurisdictions to evaluate new mothers for the risk of this condition, prenatal mental health still receives little attention as part of prenatal care [49,50]. Since antenatal depression may be more common than postnatal depression, and women with antenatal depression are more likely to be depressed postnatally [51], prenatal depression screening should be as widely practiced as postpartum screening.

One limitation of this study was the effect of multicollinearity, which is a common problem in nutrition studies, particularly those that examine the intake of nutrients from supplements as supplements include multiple nutrients and many are in similar amounts and combinations due the nutrient recommendations by health agencies. Another limitation is that the present study did not have access to serum levels of selenium or any of the other nutrients discussed in this paper, thus there is no information as to whether supplementation affected biological levels. A third limitation was that dietary (food) intake was not included in the analysis. Although dietary intake was estimated in these women at two or three trimesters during pregnancy (data which will be available at some future date), selenium intake is difficult to estimate due to variations in the food supply and the inaccuracy of nutrient databases. The high supplement intake may mitigate dietary intake in this case, and would not be likely to change our findings. We did not have lab values at present to assess anemia in our sample. Although anemia is known to be a risk factor for depression, we also know that women are routinely supplemented with iron when anemia is diagnosed during pregnancy. In this study, for women below RDA, 82.9% scored <10 EPDS, 17.1% scored ≥10 EPDS; for women above RDA, 88.5% <10 EPDS, and 11.5% were ≥10, p = 0.84 (see Table 2), indicating that there was no difference of scoring ≥10 EPDS between being above or below the RDA for iron. While we recognize this is not the ideal measure for iron associated form of anemia, it served as a proxy for iron status. Another limitation was possible reporting error associated with recall of supplement intake (e.g. dosage and frequency). Recording of brand names and labels of supplements (e.g. types and amounts of nutrients) by research assistants, as well as the use of repeated measures, helped to minimized potential reporting bias. However, we acknowledge a major limitation with the Supplement Intake Questionnaire is self-reporting; thus we did not know whether the women actually consumed their supplements as they said they did. Furthermore, if a woman missed her trimester visit then this was treated as missing data and no estimation was made. Thus, there was some degree of projection as the women were asked for information for the entire trimester at the trimester midpoint and not the end, making this a limitation to our estimates. A limitation in the analysis was that we did not assess for interaction among the nutrients due to the absence of any *a priori* rationale from the literature. In addition, as data on prenatal nutrition status was not available, we were unable to test whether the effect of prenatal micronutrient supplementation on postpartum depression varied by prenatal nutritional status. For future research, we would like to assess whether specific nutrients interact to impact postpartum depression, and also whether prenatal nutritional status modified the association of supplementation and postpartum depression.

One of the strengths of this study was the temporal relationship of supplement intake and postpartum depressive symptoms could be established. Another strength was that supplement intake was collected repeatedly over time to obtain an average usual intake of the nutrients in the prenatal period, rather than using a single measure as in most studies. A third strength was the ability to control for a large number of factors associated with postpartum depressive symptoms in our analyses because of the variables (demographic, social, lifestyle) collected prospectively.

Implications for future research include examining objective measures of pre- and postnatal micronutrient status for: 1) relations between pre/postnatal blood nutrient levels (or changes in pre/postnatal blood levels) and postpartum depression, 2) moderating effect of prenatal blood nutrient levels on the relations between prenatal micronutrient supplementation and postpartum depression, and 3) interactions amongst nutrients and their effects on postpartum depression.

## Conclusions

This study found multiple factors, including selenium intake from supplements, history of depression, and social support, are associated with the risk of postpartum depressive symptoms. The evidence suggests that there is likely no single casual factor to depression, and nutrients play an important role in the development of depressive symptoms. Thus, a future research focus on dietary supplementation with special attention to the intake of selenium in the pregnant population is warranted.

## Competing interest

All authors declare no conflict of interest, financial or otherwise.

## Authors’ contributions

BMYL carried out the data analyses and drafted the manuscript. BJK reviewed and revised the manuscript. LJM and MFG provided methodological information on the supplement intake questionnaire. CJF, ST, and LG consulted on measures of nutrition, sociodemographics, and mental health. ME provided input on statistical analysis. All authors read and approved the final manuscript.

## Pre-publication history

The pre-publication history for this paper can be accessed here:

http://www.biomedcentral.com/1471-2393/13/2/prepub
